# Low Impact of Clonal Hematopoiesis on the Determination of RAS Mutations by Cell-Free DNA Testing in Routine Clinical Diagnostics

**DOI:** 10.3390/diagnostics12081956

**Published:** 2022-08-12

**Authors:** Cristin Roma, Alessandra Sacco, Laura Forgione, Riziero Esposito Abate, Matilde Lambiase, Serena Dotolo, Monica Rosaria Maiello, Daniela Frezzetti, Guglielmo Nasti, Alessandro Morabito, Antonella De Luca, Nicola Normanno

**Affiliations:** 1 Cell Biology and Biotherapy Unit, Istituto Nazionale Tumori-IRCCS-Fondazione G. Pascale, 80131 Naples, Italy; 2 SSD Innovative Therapies for Abdominal Metastases, Istituto Nazionale Tumori-IRCCS-Fondazione G. Pascale, 80131 Naples, Italy; 3 Thoracic Medical Oncology, Istituto Nazionale Tumori-IRCCS-Fondazione G. Pascale, 80131 Naples, Italy

**Keywords:** liquid biopsy, next generation sequencing, clonal hematopoiesis

## Abstract

Targeted sequencing of circulating cell-free DNA (cfDNA) is used in routine clinical diagnostics for the identification of predictive biomarkers in cancer patients in an advanced stage. The presence of KRAS mutations associated with clonal hematopoiesis of indeterminate potential (CHIP) might represent a confounding factor. We used an amplicon-based targeted sequencing panel, covering selected regions of 52 genes, for circulating cell-free total nucleic acid (cfTNA) analysis of 495 plasma samples from cancer patients. The cfDNA test failed in 4 cases, while circulating cell-free RNA (cfRNA) sequencing was invalid in 48 cases. In the 491 samples successfully tested on cfDNA, at least one genomic alteration was found in 222 cases (45.21%). We identified 316 single nucleotide variants (SNVs) in 21 genes. The most frequently mutated gene was TP53 (74 variants), followed by KRAS (71), EGFR (56), PIK3CA (33) and BRAF (19). Copy number variations (CNVs) were detected in 36 cases, while sequencing of cfRNA revealed 6 alterations. Analysis with droplet digital PCR (ddPCR) of peripheral blood leukocyte (PBL)-derived genomic DNA did not identify any KRAS mutations in 39 cases that showed KRAS mutations at cfDNA analysis. These findings suggest that the incidence of CHIP-associated KRAS mutations is relatively rare in routine clinical diagnostics.

## 1. Introduction

The analysis of circulating cell-free DNA (cfDNA) is becoming increasingly important in the management of patients with solid tumors in an advanced stage of the disease [1]. The number of genomic biomarkers predicting a response to target therapy is continuously increasing [2,3]. Importantly, it has been estimated that over 25% of cancer patients in an advanced stage of the disease may receive a therapy based on a biomarker test [4].

The recommendations on the determination of biomarkers for precision medicine suggest that tumor tissue analysis represents the gold standard for genomic profiling. However, in cases with insufficient or inadequate tumor tissue for genetic profiling, cfDNA analysis represents a valid alternative [5]. In addition, cfDNA testing often represents the preferred approach in patients progressing on previous target therapy, because of its lower invasiveness as compared to tissue biopsy [6].

The analysis of cfDNA presents a series of limitations and problems that should not be underestimated. The amount of tumor DNA released into the bloodstream (circulating tumor DNA, ctDNA) is often limited and may, therefore, represent only a small fraction of the cfDNA isolated from peripheral blood [1,7]. The extreme dilution of ctDNA, therefore, requires the use of highly sensitive techniques for cfDNA testing [7,8]. With the increase in the number and complexity of biomarkers to be determined in some tumor types, such as carcinoma of the lung, colon or biliary tract, laboratories have progressively switched from the use of real-time PCR or emulsion PCR-based techniques to the use of targeted re-sequencing, an application of next generation sequencing (NGS).

The use of large, targeted sequencing panels can increase the error rate and also detect mutations not related to cancer cells, with particular regard to mutations associated with clonal hematopoiesis of indeterminate potential (CHIP) [9]. Most of the non-tumor DNA isolated from plasma derives from hematopoietic cells [1]. Blood cells, as is the case with all tissues, with aging accumulate mutations as a result of errors in DNA duplication and the effects of mutagenic agents [10]. When these mutations produce a proliferative advantage, they can result in clonal expansion. Recently, it has been demonstrated that the presence of clonal mutations in some genes is associated with an increased risk of the development of acute myeloid leukemia (AML) [11,12].

Mutations associated with CHIP often include genes involved in the regulation of growth and differentiation of hematopoietic cells [9,11]. However, mutations in genes that play a role in the pathogenesis of solid neoplasms have also been described [13,14]. For this reason, the presence of CHIP may represent a confounding factor for the cfDNA test in patients with solid neoplasms.

Among the CHIP-related mutations, KRAS mutations have also been described, although in a limited number of cases [13]. However, this observation raises an important diagnostic problem in light of the availability of novel drugs for patients with KRAS mutations. Recently, sotorasib was approved for the treatment of patients with non-small cell cancer (NSCLC) and KRAS p.G12C mutations [15]. The activity of sotorasib and other KRAS G12C inhibitors is currently under investigation in several malignancies carrying this specific mutation. In addition, new agents active against several RAS mutations are in clinical trials, thus making RAS mutations an increasingly relevant target for precision therapy of cancer patients [16,17,18].

Starting from these assumptions in this study, we evaluated the incidence of CHIP in the analysis of KRAS mutations in the routine diagnostic cfDNA testing in patients with advanced cancer.

## 2. Materials and Methods

### 2.1. Patients

Four hundred ninety-five plasma samples from patients with different tumor types were received in the context of our routine diagnostic workflow. The most frequent tumor type was non-small cell lung cancer (NSCLC) (374/495, 75.5%), followed by colorectal cancer (CRC) (61/495, 12.3%), cholangiocarcinoma (CCA) (21/495, 4.2%) and melanoma (18/495, 3.6%).

### 2.2. cfTNA Extraction from Plasma Samples

Peripheral blood was collected into 10.0 mL BD Vacutainer^®^ plastic tubes containing EDTA (BD Diagnostics, Milan, Italy). The plasma fraction was obtained and stored as previously described within 2 h of the blood drawing [19]. Circulating cell-free total nucleic acid (cfTNA) was extracted from 4 mL of plasma with the MagMAX Cell-Free Total Nucleic Acid Isolation Kit (ThermoFisher Scientific, Waltham, MA, USA) according to the manufacturer’s instructions. The circulating cell-free DNA (cfDNA) quantity was estimated using the Qubit 2.0 Fluorometer (Invitrogen, Waltham, MA, USA).

### 2.3. DNA Extraction from Peripheral BLOOD Leucocytes (PBL)

PBL were obtained after centrifugation of peripheral blood for 10 min at 1600× *g* at 4 °C. The supernatant containing the plasma above the PBL layer was removed into a new tube. The PBL (0.5 mL, taken from the top of the red cell pellet) was transferred into a 15 mL conical centrifuge tube with fresh, cold lysing solution, inverted for ~10 min at room temperature and centrifuged at 450× *g* for 5 min at 4 °C. The pelleted cells were suspended in 1 mL of cold PBS and centrifuged as before; this washing step was repeated twice. Finally, the isolated PBL were re-suspended in 1 mL of cold PBS. Genomic DNA (gDNA) from the PBL was isolated with the QIAamp DNA Mini Kit (Qiagen) according to the manufacturer’s instructions. The gDNA quantity was assessed using the Qubit 2.0 Fluorometer (Invitrogen). 

### 2.4. Targeted Sequencing of cfTNA

The cfTNA was analyzed with the Oncomine™ Pan-Cancer Cell-Free Assay (ThermoFisher Scientific). The panel consists of a single pool of primers to perform multiplex PCR for the sequencing of 52 genes identified as frequently mutated in multiple cancer types. This panel identified: hotspot mutations as single nucleotide variants (SNVs) and short insertions/deletions (InDels) in 44 genes; RNA alterations in 12 genes including fusion of ALK, RET and ROS1; and MET exon 14 skipping and copy number variants (CNVs) in 12 genes (see Supplementary Material for the complete list of gene alterations covered).

Twenty ng of cfTNA input, or a maximum volume of 10.4 µL per sample, was used for library construction, according to the manufacturer’s instructions, in a total volume of 13 µL. This NGS panel used Tagging Technology for rare mutations detection, assigning to each cfDNA molecule a unique molecular tag (UMI) by PCR using fusion primers that contain both gene-specific and UMI sequences. The amplified libraries were quantified with the Ion Library TaqMan Quantitation Kit (ThermoFisher Scientific). Eighty pM of each library was multiplexed and the pool was loaded on an Ion 540 chip using the Ion Chef System (ThermoFisher Scientific). Sequencing was performed on the Ion S5 XL platform and raw data were analyzed with the Torrent Suite Software v5.12.1 and the Ion Reporter Software v5.14 (ThermoFisher Scientific) using Human Genome Build 19 (hg19) as the reference. An average of 17 million reads was mapped to hg19, with the percentage of mapped reads being >90%. The coverage depth ranged from 45,000× to 70,000×, and the uniformity of each library was >98%. The median coverage for the KRAS gene was 44,910× (mean = 46,173×; the range was from 14,000× to 63,498×). The workflow for Oncomine TagSeq Pan-Cancer Liquid Biopsy w2.3 was used with default parameters. Oncomine variant annotator version 3.0 was used for variant annotation. An example of the sequencing results is available at the link http://10.5281/zenodo.6620567 (accessed on 7 June 2022).

The limit of detection (LOD) of the method correlates with the amount of cfTNA used for library preparation. To obtain 0.1% LOD (1 mutant copy in a background of 1000 wild-type copies), 20 ng of cfTNA input is required. A different LOD per sample is thus calculated based on the input cfTNA and on the coverage obtained for the sample in the region of the mutation. Each variant was verified using the IGV visualization tool (http://www.broadinstitute.org/igv/ (accessed on13 May 2022)) and in accord with the workflow of analysis described by Pasquale R. and colleagues [20].

PBL-derived DNA from five CCA samples was mechanically sheared to 150 bps to mimic the average DNA length of cfDNA before library construction [21]. Similar NGS methodology was used for DNA extracted from PBL, using an input of 20 ng.

### 2.5. ddPCR Analysis

In order to confirm the tumor origin of the KRAS variants identified by plasma testing, gDNA from 39 PBL samples was analyzed by Droplet Digital PCR (ddPCR). We employed the ddPCR KRAS G12/G13 Screening Kit #1863506, a multiplex ddPCR assay able to detect alterations in exon 12 and 13 (G12A; G12C; G12D; G12R; G12S; G12V; G13D) in the KRAS gene. Furthermore, we used the ddPCR assay KRAS Q61 Screening Kit for the following five KRAS mutations in a single well: Q61K; Q61L; Q61R; Q61H 183A > T; and Q61H 183A > C. Droplets were generated using the Bio-Rad automatic droplet generator, after which PCR amplification was performed according to the manufacturer’s instructions. At least 10,000 droplets were required for droplet generation to be considered successful. Droplets were read with the QX200 droplet digital PCR system (Bio-Rad) and analyzed using QuantaSoft software version 1.7 (Bio-Rad). The cut-off sensitivity of the ddPCR test was set at 0.1%.

## 3. Results

Our laboratory is a reference center for genomic profiling of cancer patients using either tissue or plasma samples. In the past few years, we have observed an increasing number of requests for plasma testing for patients with different tumor types. In particular, we received, in the period between August 2019 and May 2022, 495 blood samples with a request for tumor genomic profiling.

For most cases (97%), we had available the optimal volume of 4 mL of plasma from which circulating cell-free total nucleic acid (cfTNA) extraction was performed. The quantity of circulating cell-free DNA (cfDNA) extracted ranged between 0.09 and 65.9 ng/µL, with a median value of 1.27 ng/µL. We could not quantify the cfRNA levels with Qubit due to the very low levels.

The plasma-derived cfTNA was tested with the Oncomine™ Pan-Cancer Cell-Free Assay. The cfDNA test failed in 4 cases, leading to a success rate of 99.2%. However, the failure rate on circulating cell-free RNA (cfRNA) was higher, with 48/495 (9.7%) samples failing for this specific test. No sample succeeded for the cfRNA but not for the cfDNA test. However, 35/48 samples that failed at cfRNA analysis had a cfDNA concentration < 2 ng/µL, including the 4 samples with the cfDNA failure. Overall, these data suggest that low cfTNA levels were the main reason for the test failure. The histologic type of the tumors corresponding to the 491 samples that were successfully sequenced, at least on cfDNA, is indicated in Table 1.

As expected, the majority of requests were from patients carrying NSCLC (374/491, 76.2%), followed by CRC (61/491, 12.4%) and melanoma (18/491, 3.7%). However, we have observed in the past few months an increase also in the types of cancer for which the test was required, with particular regard to CCA (21 cases).

In the 491 samples that were successfully tested on cfDNA, at least 1 genomic alteration was found in 222 cases (45.21%). In particular, 140 cases carried a single genomic alteration, and 82 had multiple alterations.

**Figure 1:**
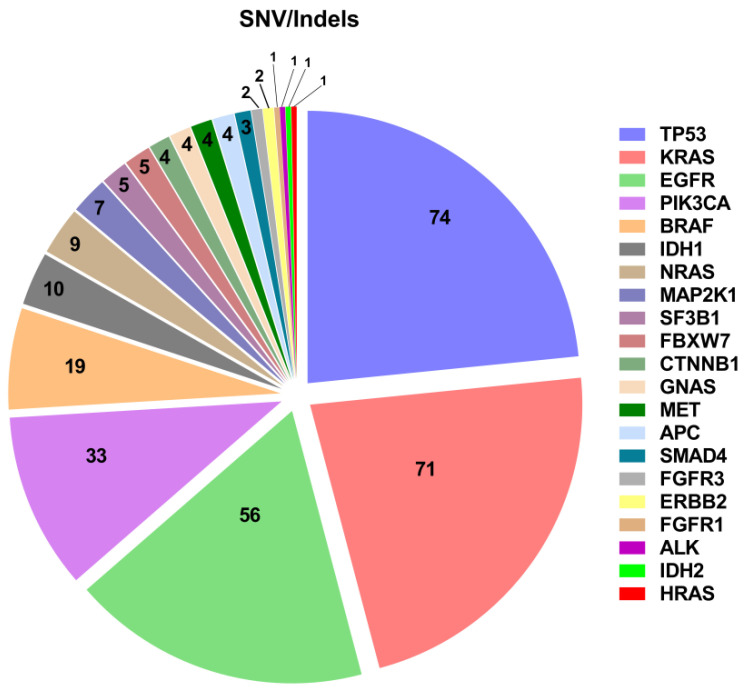
SNV identified in the cases successfully tested on cfDNA.

Overall, 316 single nucleotide variants (SNVs) and insertions/deletions (InDels) were identified in 21 different genes (Figure 1). The most frequently mutated gene was TP53 (74 variants), followed by KRAS (71), EGFR (56), PIK3CA (33) and BRAF (19). The variant allelic frequency (VAF) of the identified SNVs/InDels ranged between 0.1% and 87.5%, with a median value of 9.6 and a mean value of 16.93. The relatively high EGFR mutant rate is not surprising, due to the frequent request for liquid biopsy testing in EGFR- mutant NSCLC patients progressing on previous TKI treatment (n. 52).

**Figure 2:**
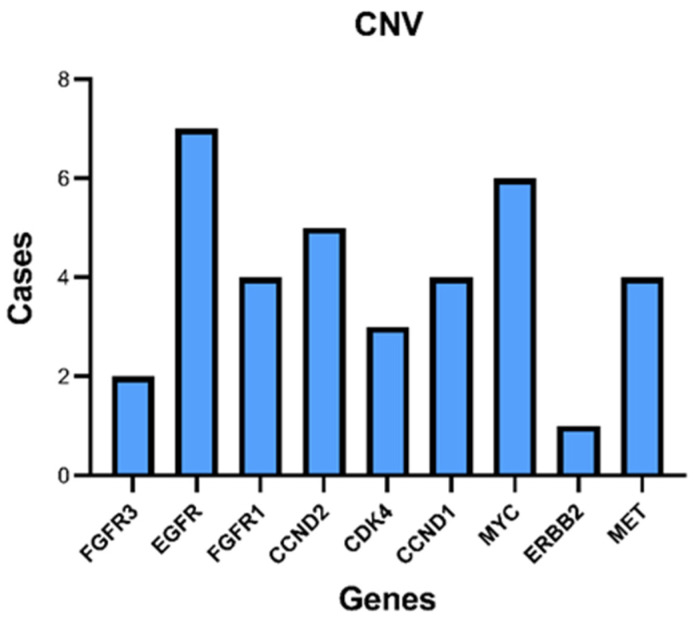
CNV detected in the plasma samples.

CNVs were found in 36 cases; all were copy number gains (Figure 2). The most frequently amplified gene was EGFR (7 cases), followed by MYC (6 cases). The 7 cases with EGFR amplification were all NSCLC.

Finally, cfRNA sequencing revealed 6 alterations, including 3 KIF5B-RET fusions, 2 EML4-ALK fusions and 1 MET exon 14 skipping alteration (Table 2).

Due to the increasing request for testing for KRAS mutations for inclusion in clinical trials and for the description in the literature of cases of KRAS mutations associated with CHIP, we aimed to confirm the tumor origin of the KRAS variants identified at cfDNA analysis by testing DNA derived from PBL. To this end, we tested by ddPCR genomic DNA isolated from PBL of 39 KRAS-positive cases on cfDNA (28 NSCLC, 9 CRC and 2 CCA). The mean age of this subgroup of patients was 67.46 years (median = 68 years; min value = 44 years; max value = 90 years), similar to the mean age of the whole patient population of 67.3 years (median = 67 years; min value = 18 years; max = 88 years; Student’s t-test *p* value = 0.9369). The VAF of KRAS variants among this group varied between 0.1% and 37.3%, with a median value of 3.55 and a mean value of 7.22 (Table 3).

Testing PBL-derived genomic DNA with ddPCR did not reveal KRAS variants in any of the cases (Table 3). In five cases, we analyzed the gDNA from PBL with the same panel used for cfTNA testing, and no KRAS variant was identified (data not shown). Finally, for nine cases, we had available also the matched tumor tissue. In all cases, we found in the tumor tissue the same KRAS variant identified in the cfDNA (Table 3).

## 4. Discussion

The use of cfDNA analysis is becoming increasingly important in the clinical management of patients with advanced malignancies [1,7,22]. The analysis of both cfDNA and cfRNA (cfTNA) has to take into account all the different limitations that can affect the results of the test. In particular, the analytical sensitivity of the test, the amount of cfTNA that can be isolated from the peripheral blood, the type of genomic alteration investigated, the tumor burden and localization of the tumor sites, and the timing of blood sampling (the first diagnosis versus progression after the first-line therapy) are all factors that can affect the results of the cfTNA testing.

Several studies have demonstrated the reliability of NGS-based methods for the genomic profiling of cancer, starting with cfDNA [20,23,24]. Above all, the cfDNA test is increasingly being used to make treatment decisions [25,26,27].

In this paper, we describe the results of cfTNA testing with targeted sequencing in the context of a referral center diagnostic routine. Although the analysis was successful in over 99% of cases, at least for cfDNA, genomic alterations were detected in only about 45% of successfully analyzed cases. The frequency of cases with genomic alterations varies significantly among the different studies, with some papers reporting up to 86% of the samples carrying a somatic alteration [23]. The frequency of variants detected in plasma testing is affected by many variables, including the genomic regions covered and the sensitivity of the test. The most frequently altered gene found in our study was TP53, followed by KRAS, EGFR, PIK3CA and BRAF. The frequency and the number of alterations found are in line with those of previous reports that describe the analyses of liquid biopsy samples using an NGS approach. Moreover, the genomic alterations identified in our study reflect the genomic landscape identified by tissue testing of tumors of the same histological type [20,23,24]. In this respect, the assay that we used in our routine clinical workflow covers selected regions in 52 genes, a lower number as compared to other studies [23]. In addition, the sensitivity of the assay is directly related to the input cfTNA. With a median cfDNA value of 1.27 ng/µL and a maximum volume of input cfTNA of 10.4 µL, in >50% of the cases, a suboptimal <20 ng of cfDNA amount was sequenced. Indeed, levels of ctDNA can affect the possibility to detect different types of variants [28]. Although it was not possible to quantify the cfRNA content of the samples, it is likely that cfRNA levels were, as well, non adequate. The low quantity of nucleic acids isolated from the plasma samples might be related to different factors, including the tumor burden and the timing of blood sampling with respect to the disease phase (patients in response to treatment versus oligo-progression versus rapid progression of the disease). 

However, it should be emphasized that numerous actionable alterations have been identified in this study, allowing for important therapeutic decisions in patients who would not have had other treatment options.

Sequencing of cfRNA revealed the presence of alterations in some of the patients we tested. We cannot estimate the sensitivity of the test to detect fusions, due to the lack of cases with matched tissue samples. However, the high failure rate suggests that cfRNA sequencing has a relatively low sensitivity in detecting RNA-associated alterations. Further investigation will be needed to define the suitability of this approach for clinical practice.

In a scenario of the increasing therapeutic relevance of cfDNA testing, it appears essential to guarantee the absolute specificity of the cfDNA test, especially in the identification of actionable mutations. Several studies have described a high incidence of CHIP-associated mutations in cfDNA analysis, with >50% of the mutations detected in the cfDNA due to this phenomenon [29]. Analysis of the association of CHIP with the cfDNA test found a clear correlation between CHIP and the age of the patients, as expected [11]. However, the frequency of identification of CHIP-associated mutations also depends on the width and sensitivity of the panels used [11].

The tumor type for which the use of liquid biopsy is most frequent is NSCLC, due to the limited quantity and quality of the material available for genomic profiling in a relatively high percentage of patients [8]. NSCLC is also the first cancer for which the use of a drug directed against a specific KRAS mutation, p.G12C, has been approved [15]. However, KRAS inhibitors are being explored in several tumor types since KRAS mutations are among the most frequent genomic alterations causing cancer [16,30,31]. 

Some studies have reported the possible association of KRAS mutations with CHIP, albeit at a low frequency. Specifically, Hu et al. [13], described the presence of KRAS mutations in two NSCLC patients who also carried EGFR mutations. In both patients, analysis of DNA extracted from the PBL confirmed a clonal hematopoiesis origin. However, the coexistence of EGFR and KRAS mutations has been demonstrated in rare cases by tumor tissue testing, thus excluding that this phenomenon is only related to CHIP [32].

Our study demonstrates that, in the context of a cfDNA testing diagnostic service, the incidence of KRAS mutations associated with clonal hematopoiesis is a relatively rare occurrence. These data confirm the reliability of the cfDNA test for the selection of patients for treatment with KRAS inhibitors. Of course, our conclusions cannot be extrapolated to other testing methods, which should carry out a similar validation.

In conclusion, our data suggest that cfTNA sequencing is feasible in the routine clinical scenario. The sensitivity of the test is affected by the quantity of nucleic acids that can be isolated from the peripheral blood. CHIP does not seem to represent a relevant issue for KRAS mutation testing in cfDNA.

## Figures and Tables

**Table 1:** Tumor types of plasma samples successfully sequenced at least for cfDNA.

Tumor Type	N° Cases
NSCLC	374
Colorectal	61
Melanoma	18
Cholangiocarcinoma	21
Pancreas	4
Breast	3
Vater’s papilla	2
Hepatocarcinoma	2
Ovarian Cancer	2
Stomach	1
Bladder cancer	1
Parathyroid cancer	1
Cardias adenocarcinoma	1

**Table 2:** RNA alterations detected.

Tumor Type	Gene	Genomic Alterations	N. Cases
NSCLC	RET	KIF5B-RET.K23R12.COSF1234	2
NSCLC	RET	KIF5B-RET.K15R12.COSF1232.1	1
NSCLC	MET	MET-MET.M13M15	1
NSCLC	ALK	EML4-ALK.E13A20.COSF408.2	1
NSCLC	ALK	EML4-ALK.E6aA20.AB374361	1

**Table 3:** Analysis of PBL-derived genomic DNA for KRAS mutations.

N.	Tumor Type	KRAS Variant in cfDNA	ddPCR on PBL	Matched Tissue
10502	NSCLC	p.G12D; c.35G > A (7.8%)	WT	NA
10640	NSCLC	p.G12D; c.35G > A (8.6%)	WT	NA
10876	NSCLC	p.G12A; c.35G > C (2.8%)	WT	NA
10951	NSCLC	p.G12D; c.35G > A (1.8%)	WT	NA
10996	NSCLC	p.G12A; c.35G > C (3.5%)	WT	NA
11009	NSCLC	p.G12C; c.34G > T (14.2%)	WT	NA
11023	NSCLC	p.G12C; c.34G > T (6.2%)	WT	NA
11032	NSCLC	p.Q61H; c.183A > T (2.8%)	WT	p.Q61H; c.183A > T (28.8%)
11139	NSCLC	p.G12C; c.35G > A (9.1%)	WT	NA
11193	NSCLC	p.G12V; c.35G > T (3.4%)	WT	NA
11239	NSCLC	p.G12C; c.34G > T (2.2%)	WT	p.G12C; c.34G > T (23.6%)
11332	NSCLC	p.G12V; c.35G > T (20.9%)	WT	NA
11341	NSCLC	p.G12V; c.35G > T (23.8%)	WT	p.G12V; c.35G > T (23.7%)
11503	NSCLC	p.G12V; c.35G > T (17.6%)	WT	NA
11581	NSCLC	p.G12V; c.G35T (1.3%)	WT	NA
11598	NSCLC	p.G12D; c.G35A (1.2%)	WT	NA
11672	NSCLC	p.G12C; c.34G > T (0.8%)	WT	NA
11715	NSCLC	p.G12C; c.34G > T (33%)	WT	p.G12C; c.34G > T (5.6%)
11998	NSCLC	p.Q61H; c.183A > C (6.1%)	WT	NA
13366	NSCLC	p.G12V; c.35G > T (1.4%)	WT	NA
13272	NSCLC	p.G13D; c.38G > A (0.2%)	WT	NA
13316	NSCLC	p.Q61H; c.183A > T (0.7%) p.G12V; c.35G > T (0.9%)	WT	NA
10527c	NSCLC	p.G12R; c.34G > C (3.2%)	WT	p.G12R; c.34G > C (14.4%)
13244	NSCLC	p.G12V; c.35G > T (6.9%)	WT	p.G12V; c.35G > T (61.6%)
12951	NSCLC	p.G12V; c.35G > T (3.7%)	WT	p.G12V; c.35G > T (47.9%)
12642	NSCLC	p.Q61L; c.182A > T (1.4%)	WT	p.Q61L; c.182A > T (17.6%)
12145	NSCLC	p.G12S; c.34G > A (1.7%)	WT	NA
12253	NSCLC	p.G12C; c.34G > T (7%)	WT	p.G12C; c.34G > T (70.9%)
10366	CRC	p.G13D; c.38G > A (13%)	WT	NA
10745	CRC	p.G12D; c.35G > A (0.3%)	WT	NA
10795	CRC	p.G12D; c.35G > A (14.5%)	WT	NA
10985	CRC	p.G13D; c.38G > A (0.12%)	WT	NA
10314	CRC	p.G12D; c.35G > A (5.1%)	WT	NA
11124	CRC	p.G12S; c.34G > A (2.9%)	WT	NA
11505	CRC	p.G13D; c.38G > A (37.3%)	WT	NA
11576	CRC	p.G12A; c.G35C (10.4%)	WT	NA
10963	CRC	p.G12C; c.34G > T (6.9%)	WT	NA
10478	CCA	p.G12D; c.35G > A (3.6%)	WT	NA
10762	CCA	p.G12D; c.35G > A (0.5%)	WT	NA

Abbreviations: NSCLC, non-small cell lung cancer; CRC, colorectal cancer; CCA, cholangiocarcinoma; WT, wild-type; NA, not analyzed; ddPCR, droplet digital PCR; PBL, peripheral blood leukocyte.

## Data Availability

An example of the sequencing results is available at the link http://10.5281/zenodo.6620567 (accessed on 7 June 2022).

## Supplementary Materials

The following supporting information can be downloaded at: https://www.mdpi.com/article/10.3390/diagnostics12081956/s1.
