# Supplementary material for: Low Impact of Clonal Hematopoiesis on the Determination of RAS Mutations by Cell-Free DNA Testing in Routine Clinical Diagnostics
Source: Diagnostics (Basel). 2022 Aug 12;12(8):1956. doi: 10.3390/diagnostics12081956 (PMC9406879; doi:10.3390/diagnostics12081956)
Supplement: Supplementary file 1 [file diagnostics-12-01956-s001.zip › diagnostics-1787441-supplementary.pdf]

Targeted sequencing of cfTNA

The Oncomine™ Pan-Cancer Cell-Free Assay (ThermoFisher Scientific) was used to targeted sequencing of cfTNA from 495 plasma samples. This panel consists of a single pool of primers to perform multiplex PCR for sequencing of the 52 genes. It is able to detect single nucleotide variants (SNVs) and short insertions and deletions (InDels) in the following hotspot genes: AKT1, ALK, AR, ARAF, BRAF, CHEK2, CTNNB1, DDR2,EGFR, ERBB2, ERBB3, ESR1, FGFR1, FGFR2, FGFR3, FGFR4, FLT3, GNA11, GNAQ, GNAS, HRAS, IDH1, IDH2, KIT, KRAS, MAP2K1, MAP2K2, MET, MTOR, NRAS, NTRK1, NTRK3, PDGFRA, PIK3CA, RAF1, RET, ROS1, SF3B1, SMAD4, SMO, APC, FBXW7, PTEN, TP53. Moreover, this panel can reveal copy number variants (CNVs) in the following genes: CCND1, CCND2, CCND3, CDK4, CDK6, EGFR, ERBB2, FGFR1, FGFR2, FGFR3, MET, MYC. Finally, fusions in ALK, BRAF, ERG, ETV1, FGFR1, FGFR2, FGFR3, MET, NTRK1, NTRK3, RET, ROS1 genes and the MET exon 14 skipping can be detected using this panel.
